# Genome-wide analysis of the role of GlnR in *Streptomyces venezuelae *provides new insights into global nitrogen regulation in actinomycetes

**DOI:** 10.1186/1471-2164-12-175

**Published:** 2011-04-04

**Authors:** Steven T Pullan, Govind Chandra, Mervyn J Bibb, Mike Merrick

**Affiliations:** 1 Department of Molecular Microbiology, John Innes Centre, Norwich Research Park, Norwich, Norfolk NR4 7UH, UK; 2 Institute of Genetics, School of Biology, University of Nottingham, University Park, Nottingham NG7 2RD, UK

## Abstract

**Background:**

GlnR is an atypical response regulator found in actinomycetes that modulates the transcription of genes in response to changes in nitrogen availability. We applied a global *in vivo *approach to identify the GlnR regulon of *Streptomyces venezuelae*, which, unlike many actinomycetes, grows in a diffuse manner that is suitable for physiological studies. Conditions were defined that facilitated analysis of GlnR-dependent induction of gene expression in response to rapid nitrogen starvation. Microarray analysis identified global transcriptional differences between *glnR*^+ ^and *glnR *mutant strains under varying nitrogen conditions. To differentiate between direct and indirect regulatory effects of GlnR, chromatin immuno-precipitation (ChIP) using antibodies specific to a FLAG-tagged GlnR protein, coupled with microarray analysis (ChIP-chip), was used to identify GlnR binding sites throughout the *S. venezuelae *genome.

**Results:**

GlnR bound to its target sites in both transcriptionally active and apparently inactive forms. Thirty-six GlnR binding sites were identified by ChIP-chip analysis allowing derivation of a consensus GlnR-binding site for *S. venezuelae*. GlnR-binding regions were associated with genes involved in primary nitrogen metabolism, secondary metabolism, the synthesis of catabolic enzymes and a number of transport-related functions.

**Conclusions:**

The GlnR regulon of *S. venezuelae *is extensive and impacts on many facets of the organism's biology. GlnR can apparently bind to its target sites in both transcriptionally active and inactive forms.

## Background

The effective assimilation and utilisation of nitrogen are challenges shared by all bacterial species. The mechanisms of regulation of nitrogen metabolism vary greatly but in most organisms overall control is mediated by a global transcriptional regulator [1-3]. GlnR is one such transcriptional regulator belonging to the OmpR winged helix-turn-helix family. It plays a key regulatory role in the expression of genes involved in nitrogen metabolism in several actinomycetes, including *Streptomyces coelicolor *[4], *Amycolatopsis mediterranei *[5], *Mycobacterium smegmatis *[6] and the human pathogen *Mycobacterium tuberculosis *[7].

GlnR was first identified in *S. coelicolor *by its ability to restore wild-type growth to a glutamine auxotroph [8]. It was subsequently shown to activate expression of genes involved in ammonium assimilation, including *glnA *and *glnII *that encode glutamine synthetase isoenzymes GSI and GSII, respectively, and *amtB *that encodes an ammonium transporter [9]. Co-transcribed with *amtB *are *glnK *and *glnD*, which encode an unusually modified (adenylylated) PII protein and its partner adenylyltransferase, respectively [10]. A second OmpR-like regulator, highly similar to GlnR, is encoded by *glnRII*, which lies adjacent to *glnII*. GlnRII binds to the same promoter sequences as GlnR, but its role in nitrogen metabolism is not known [9].

The range of genes regulated by GlnR in *S. coelicolor *was extended by the work of Tiffert *et al. *[11] initially using a bioinformatic approach. By searching for promoters containing a consensus GlnR-binding sequence and verifying GlnR binding activity *in vitro*, they identified 10 new GlnR targets. These included genes involved in the utilisation and assimilation of various nitrogen sources, such as nitrite and urea, as well as multiple genes with uncharacterised functions. Recently *S. coelicolor nasA*, encoding nitrate reductase, was also found to be regulated by GlnR through an interaction with a promoter sequence somewhat different from those previously association with GlnR binding [12]. Thus while a predictive bioinformatic approach can be extremely powerful, and has indeed provided considerable insight into the GlnR regulon of *S. coelicolor*, it is by no means comprehensive. The existence of unusual GlnR binding sequences, such as that found upstream of *nasA*, implies that there may be other, as yet undiscovered, GlnR target genes. The recent demonstration that the expression of *glnR *and of some of the GlnR-regulated genes of *S. coelicolor *is subject to repression by PhoB, the response regulator component of the phosphate sensing system [13], highlights the cross-talk that can occur between regulatory systems involved in the global co-ordination of primary metabolism. Thus, the regulatory effects of GlnR may extend beyond primary nitrogen metabolism, and indeed a recent proteomic analysis of the GlnR-mediated response to nitrogen limitation in *S. coelicolor *also came to this conclusion [14]. Interestingly, the GlnR orthologue of *A. mediterranei *is involved in the regulation of rifamycin production and its heterologous expression in *S. coelicolor *had marked effects on secondary metabolism, causing precocious production of undecylprodigiosin and inhibiting actinorhodin production [15]. Such observations suggest that GlnR may play a role in the regulation of secondary metabolism in other actinomycetes. Intriguingly, in *Streptomyces venezuelae *chloramphenicol production is influenced by the availability of both nitrogen and carbon [16].

The aim of this study was to apply a global *in vivo *approach to the identification of GlnR and GlnRII-regulated genes. Global transcriptional profiles of *glnR *and *glnRII *mutants were compared to that of the wild-type strain during growth in varying conditions of nitrogen availability to identify changes in gene expression dependent on either regulator. In addition, global analysis of GlnR-DNA interactions was performed using chromatin immunoprecipitation coupled to microarray analysis of enriched target sites (ChIP-chip).

*S. venezuelae *was chosen for this study for several reasons. It grows in a diffuse and homogenous manner in a variety of liquid media, and in some sporulates to near completion [17]. Such growth characteristics reduce the physiological heterogeneity inevitably associated with the irregularly sized mycelial clumps observed in most actinomycete liquid cultures. This, together with the availability of *S. venezuelae *microarrays suitable for both transcriptional and ChIP-chip analyses, made this organism an extremely attractive system for such physiological studies.

## Results

### GlnR and GlnRII in *S. venezuelae*

The *S. venezuelae *genome sequence [GenBank Accession No. FR845719] encodes predicted homologues of GlnR and GlnRII, both of which occur in regions with a high degree of synteny with the respective chromosomal locations in *S. coelicolor*. *S. venezuelae *GlnR and GlnRII show 80.6% and 67% identity, respectively, to their *S. coelicolor *homologues.

### Defining conditions for induction of the GlnR regulon

To aid in the accurate interpretation of global transcriptional data, a minimal Evans medium [18] was used that precisely defined the sources of all nutrients, providing a clear physiological perspective for data analysis. *S. venezuelae *showed reproducible, vigorous growth in Evans medium containing 30 mM ammonium chloride as nitrogen source. A maximum growth rate of 0.27 h^-1 ^was achieved during exponential phase and consistently dispersed mycelium throughout growth was verified microscopically (data not shown).

**Figure 1:**
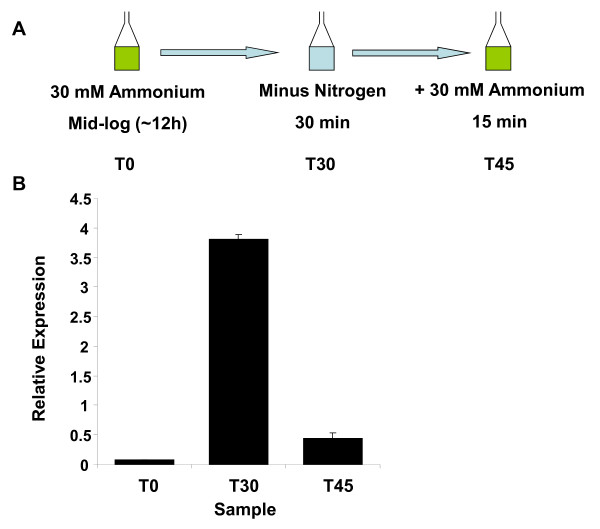
***amtB *is induced during nitrogen starvation, and repressed by the presence of ammonium chloride**. (A) Time course and conditions used in the majority of experiments in this study. (B) qRT-PCR measurement of *amtB *transcript levels over the time course. Expression levels under each condition were normalised to levels of the mRNA of the major vegetative sigma factor gene, *hrdB*. Data was collected from three independent biological samples with triplicate technical repeats of each qPCR reaction. Error bars represent standard deviation.

To determine conditions suitable for global transcriptional analysis, qRT-PCR was used to examine the effect of various growth conditions on transcription of the well-characterised GlnR target *amtB *[9]. Optimal induction of *amtB *occurred in response to a switch from mid-log growth in Evans containing 30 mM ammonium chloride to Evans medium completely lacking a nitrogen source for a 30 min period. This induction was almost entirely reversed by the exogenous addition of 30 mM ammonium chloride to the starved culture for a 15 min period. Transcript levels of *amtB*, assessed by quantitative reverse-transcriptase polymerase chain reaction (qRT-PCR), were induced 53-fold by nitrogen starvation and this level was reduced 9-fold by ammonium addition (Figure 1).

### Global transcriptional changes in response to nitrogen status

RNA samples harvested from three time points (T_0 _- prior to N starvation, T_30 _- 30 mins after N starvation, and T_45 _- 15 mins after addition of ammonium) were used to produce cDNA for hybridisation to Affymetrix GeneChips. Three separate biological replicates of this experiment provided a global transcriptional profile of gene expression under conditions that had a marked effect on *amtB *expression. The microarray data confirmed the pattern of *amtB *expression measured using qRT-PCR, i.e. the gene was strongly induced (76-fold) upon N starvation and was repressed (15-fold) by exogenous addition of ammonium (Additional file 1). To identify genes with a similar pattern of gene expression, data were filtered to include those genes that were both induced >2 fold in response to N-limitation and repressed by >2 fold in response to the exogenous addition of ammonium. In total 116 genes (Additional file 2) fitted these criteria. A truncated list of the twenty genes showing the largest degree of induction upon N-starvation is given in Table 1. The fact that the majority of inductions observed are not equally matched by the repression following the re-addition of ammonium may be due to the persistence of mRNA after transcriptional activation has been abolished. The majority of genes that are highly induced are so because the level of expression prior to induction is very low. Therefore a relatively low number of persisting transcripts can have a large effect on the change in expression level when viewed in terms of a fold change.

**Table 1:** The 20 genes showing the greatest fold induction upon nitrogen starvation, that are also repressed by ammonium

Gene ID	*S. coelicolor *homologue	Annotation	Fold Induction in WT at T30	Fold Repression in Wt at T45
				
Sven_5279	SCO5583	ammonium transporter *amtB*	76.4	15.0
Sven_5281	SCO5585	PII uridylyltransferase *glnD*	26.0	8.3
Sven_2720	SCO2958	putative transcriptional regulator	21.6	6.0
Sven_5280	SCO5584	nitrogen regulatory protein PII *glnK*	16.0	6.8
Sven_2606	SCO2816	conserved hypothetical protein	15.4	3.9
Sven_0745	SCO1118	putative integral membrane protein	13.6	9.9
Sven_4809	SCO5163	unknown	12.5	3.7
Sven_0933	-	putative cholesterol esterase	11.7	7.2
Sven_6300	SCO6803	putative acetyltransferase	9.8	2.9
Sven_4564	SCO4896	putative transport integral membrane protein	9.2	7.3
Sven_1595	SCO1963	putative integral membrane export protein	8.7	2.2
Sven_3456	SCO6809	putative integral membrane transport protein	7.9	6.2
Sven_1523	-	unknown	7.7	4.5
Sven_2474	SCO5348	putative excisionase	7.4	2.7
Sven_3334	SCO3564	putative Na^+^/H^+ ^antiporter	7.3	4.6
Sven_0739	SCO1109	putative oxidoreductase	7.2	4.4
Sven_3030	SCO3185	putative Na^+^/H^+ ^antiporter	7.1	4.1
Sven_0867	SCO1293	putative acetyltransferase	6.7	5.2
Sven_1874	SCO2210	glutamine synthetase II *glnII*	6.5	3.6
Sven_3001	SCO3167	putative TetR-family transcriptional regulator	6.4	7.5
				

Full list of genes induced >2 are shown in Additional file 2. Fold induction is calculated as the ratio signal T30/signal T0. Fold repression is the ratio signal T30/signal T45. Values given are the average of three hybridisations using cDNA generated from three independent biological experiments. Annotations taken from StrepDB database (http://strepdb.streptomyces.org.uk)

This list includes a number of genes, e.g. *amtB *and *glnII*, previously demonstrated to respond to changes in nitrogen status in a GlnR-dependent manner in *S. coelicolor *[11]. Other genes that showed a similarly dramatic response upon microarray analysis, such as Sven_2720 encoding a putative transcriptional regulator fused to a uroporphyrinogen III synthase-like domain (homologous to SCO2958), were therefore also strong candidates for GlnR regulation. However, the stringent N-limitation conditions used in these experiments could also have induced expression of genes responding to reduced growth rate or of those involved in general starvation responses. Indeed this might be indicated by the induction of several uncharacterised transport systems (Table 1). To identify responses that were GlnR-mediated, a comparative transcriptional approach was taken using *glnR *and *glnRII *mutant strains.

### Construction and analysis of mutant strains

To identify which of the transcriptional changes occurring over the time-course were GlnR- or GlnRII-dependent, experiments were performed with mutant strains lacking each of the regulatory proteins. Mutants were generated using PCR-targeting [19], which replaced the entire coding region of each gene with an apramycin resistance cassette. Phenotypic analysis of growth on solid Evans media with ammonium, glutamine, glutamate, nitrate, asparagine or casamino acids as nitrogen source revealed no gross phenotypic differences between the wild-type and *glnRII *mutant strain M1245 on any nitrogen source tested. The *glnR *mutant strain M1246 showed a slight reduction in growth rate on each nitrogen source, but the only one on which it failed to grow entirely was nitrate, consistent with previous observations in *S. coelicolor *[11].

### Effects of *glnR *or *glnRII *mutation on global transcription

Of the 116 genes in the wild-type strain that showed a significant induction during N-starvation, and repression upon the addition of exogenous ammonium chloride (Additional file 2), 70 failed to respond to identical conditions in the *glnR *mutant (Additional file 3). GlnR-dependent genes induced >5 fold by N-starvation in the wild-type strain but not in the *glnR *mutant are listed in Table 2.

**Table 2:** Genes induced >5 fold by nitrogen starvation and repressed by ammonium in the wild-type strain, but non-responsive in the *glnR *mutant strain

Gene ID	*S. coelicolor *homologue	Annotation
		
Sven_5279	SCO5583	ammonium transporter, *amtB*
Sven_5281	SCO5585	PII uridylyltransferase, *glnD*
Sven_2720	SCO2958	putative transcriptional regulator
Sven_2606	SCO2816	conserved hypothetical protein
Sven_6300	SCO6803	putative acetyltransferase
Sven_4564	SCO4896	putative integral membrane protein
Sven_1595	SCO1963	putative integral membrane export protein
Sven_2474	SCO5348	putative excisionase
Sven_3334	SCO3564	putative Na^+^/H^+ ^antiporter
Sven_0867	SCO1293	c-terminal homology to acetyltransferase
Sven_1874	SCO2210	glutamine synthetase II *glnII*
Sven_5427	SCO5772	putative cysteine dioxygenase
Sven_1172	SCO1578	acetylglutamate kinase
Sven_3383	-	putative PIN domain containing protein
Sven_0779	-	putative permease
Sven_6135	-	putative major facilitator super family transporter
Sven_2419	SCO2636	unknown
Sven_1169	SCO1572	putative secreted protein
Sven_4152	SCO4337	putative integral membrane efflux protein
Sven_1173	SCO1579	putative glutamate N-acetyltransferase
Sven_2176	SCO2362	unknown
		

A full list of genes induced >2 fold is shown in Additional file 3.

In contrast to the major transcriptional changes observed in the *glnR *mutant, deletion of *glnRII *did not significantly perturb the expression of any genes noted previously to respond to changes in nitrogen availability (Table 1). 165 genes showed a significant change in expression upon nitrogen starvation when the *glnRII *mutant was compared to the wild-type strain. However most of these genes exhibited considerably smaller fold changes than those caused by *glnR *mutation. Only 7 genes showed a difference of greater than 5-fold, with the highest being a 13-fold increase in expression of Sven_1878 encoding a putative integral membrane protein of unknown function. None of these genes were homologous to genes implicated in nitrogen metabolism in other systems. In an attempt to determine a physiological role for GlnRII, we used the motif-finding program MEME [20] (http://meme.sdsc.edu) to search for a sequence motif within the upstream regions of genes whose transcriptional profile was altered in the *glnRII *mutant. No common feature was detected.

### Direct or indirect regulation by GlnR

While the 70 genes in Additional file 3 exhibited a marked GlnR-dependent change in transcription in response to altered nitrogen status, these genes could be directly regulated by GlnR or could be regulated by factors downstream of GlnR in a possible regulatory cascade. Such indirect regulation was also recently proposed by Tiffert *et al. *[14]. Of the 70 genes, eight encode putative transcriptional regulatory proteins and another encodes an RNA polymerase sigma factor, and changes in the levels of these proteins would likely affect the expression of their target genes. Equally, any changes in the levels of metabolites that occur during growth in the absence of GlnR could post-translationally affect the activities of other transcriptional regulators and thereby also alter the transcriptional profile. This is a fundamental limitation of transcriptional profiling. Hence, to identify genes directly regulated by GlnR, i.e. by interaction of the protein with their promoter regions, a ChIP-chip approach was taken.

### GlnR ChIP-chip

**Figure 2:**
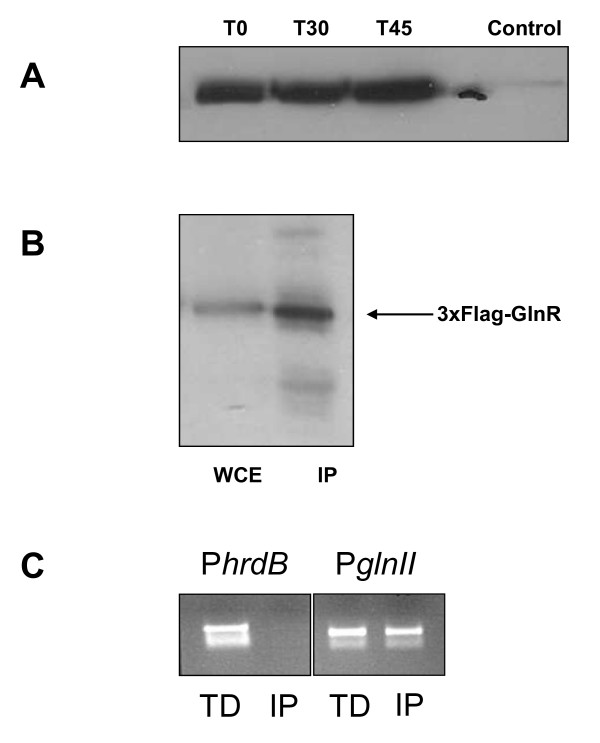
**Verification of detection, immunoprecipitation and target promoter enrichment using anti-Flag antibody and Flag-tagged GlnR**. (A) Western blot showing the presence of GlnR-Flag protein at each time-point described in Figure 1A; (B) the presence of GlnR-Flag in immuno-precipitates (IP) from whole-cell-lysates (WCE) was verified by Western blotting; (C) The presence of the promoter region of the GlnR target *glnII*, and absence of the promoter of the non-GlnR-regulated *hrdB *in immunoprecipitated samples (IP) was confirmed by PCR; total DNA (TD) was used as a control template.

For ChIP experiments, a construct was made in which GlnR carried a C-terminal 3xFlag-epitope tag. The construct (pIJ12248) allows the expression of GlnR-Flag under the control of the native *glnR *promoter from a plasmid integrated at the phage φBT1 attachment site [21]. The construct was transferred into the *glnR *mutant M1246 by conjugation to create M1255. Detection of GlnR-Flag at all three stages of the time course experiment was confirmed by Western blotting (Figure 2A), and functionality of the fusion protein was confirmed by the restoration of growth of M1256 on nitrate as sole nitrogen source (data not shown).

**Figure 3:**
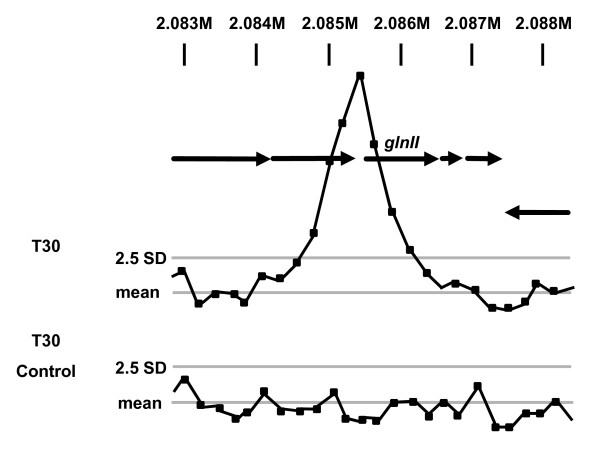
**ChIP-chip analysis of GlnR binding to the *glnII *promoter region**. Plotting the ratio of IP DNA to control total DNA across the genome for the GlnR-Flag IP at T30 and the control (not expressing Flag-tagged GlnR) at the same time point (below), indicates a peak in the *glnII *promoter region that is specific to the GlnR-Flag IP, indicating a true GlnR binding site. Light grey lines indicate the mean ratio of all points and 2.5 standard deviations from the mean. Similar peaks were observed for all those genes listed in Table 3.

Immunoprecipitation (IP) trials, followed by Western blotting, demonstrated enrichment of GlnR-Flag protein following IP (Figure 2B). DNA eluted from the precipitated protein complexes was confirmed by PCR to include the promoter region of *glnII*, a defined GlnR target, but not that of *hrdB *which encodes the major sigma factor of *S. venezuelae *and which did not respond to changes in nitrogen status in the microarray experiments (Figure 2C). Having confirmed enrichment of GlnR target sequences, IP DNA was labelled with Cy-3 and hybridised to fully-tiled whole genome arrays of *S. venezuelae *(Oxford Gene Technology). Samples were taken at each of the three time points used for transcriptional analysis, and hybridised along with Cy-5-labelled control samples of total DNA from the same time point that had not been subjected to immunoprecipitation. Plotting the Cy3/Cy5 ratio across the chromosome allowed identification of peaks corresponding to GlnR-associated sites. Peaks were considered significant when at least 2 consecutive probes had a Cy3/Cy5 ratio greater than 2.5 standard deviations above the mean of all signals. This analysis was carried out in duplicate on separate biological samples. As a further control, immunoprecipitation was carried out using the wild-type strain carrying the empty Flag vector (pIJ10500) and therefore not making the GlnR-Flag fusion protein. Peaks that were also present in this sample were considered to be artefacts. Figure 3 shows an example of a GlnR-dependent binding site located within the *glnII *promoter region.

### GlnR binding is promoted by nitrogen starvation, but is not abolished by ammonium addition

**Figure 4:**
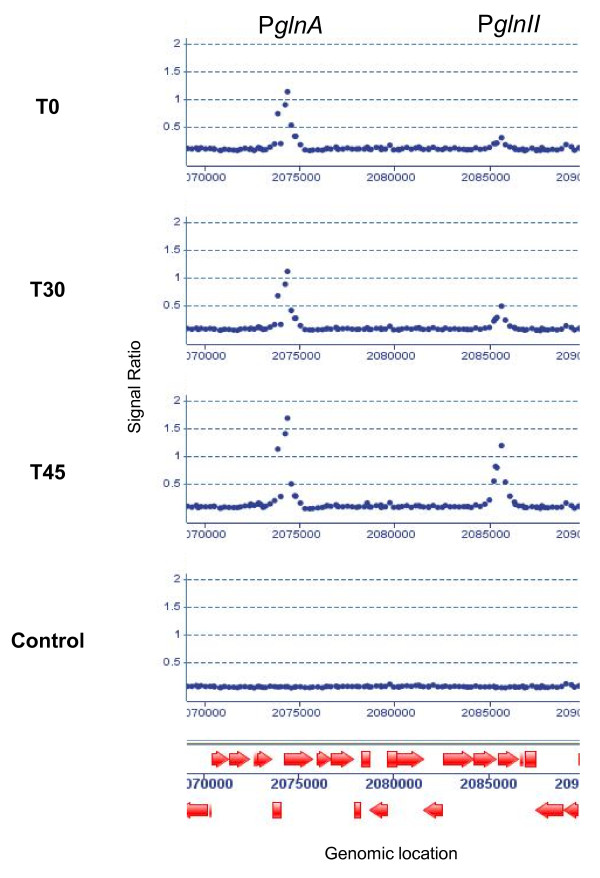
**GlnR-binding to the *glnA *and *glnII *promoters over the time course**. The ratio of IP DNA to control total DNA over the genomic region encompassing the genes encoding GSI and GSII (*glnA and glnII*) reveals a peak within the *glnA *promoter region at all time points, indicating that GlnR is constantly associated with the promoter. The *glnII *promoter shows significantly higher peaks at T30 and T45, indicating that GlnR binding is promoted by nitrogen starvations, but is not relieved by the addition of exogenous ammonium chloride.

At time point T_0 _(Figure 1a), representative of mid-log phase in the presence of 30 mM ammonium chloride, a single significant GlnR-dependent peak was identified that corresponded to the promoter region of *glnA *(Figure 4). This low level of binding at target promoters is consistent with the transcriptional data from both microarrays and qRT-PCR analysis that shows low transcriptional activity from GlnR-target promoters under conditions of nitrogen sufficiency. Binding to the *glnA *promoter at this time is also consistent with relatively higher levels of *glnA *transcription at T_0 _and lower levels of induction upon nitrogen starvation when compared with other target genes, such as *glnII *or *amtB*. Such regulation correlates well with a role for GSI as the "house-keeping" glutamine synthetase, which is active during times of nitrogen sufficiency, whilst GSII is induced during conditions of starvation. Expression of GSII-type isoenzymes in low nitrogen levels has also been demonstrated in plant symbiotic *Rhizobia *species [22].

After 30 minutes of nitrogen starvation (T_30_) many more GlnR peaks were observed. The most highly enriched regions were those containing the *glnA *and *glnII *promoters. The *amtB *promoter was also highly represented in the IP material. This coincides with the maximal levels of expression of these genes during the time course. However, by T_45 _exposure of the starved cultures to exogenously added ammonium chloride did not abolish the GlnR binding detected at many promoter sites during N starvation, despite the fact that by T_45 _many of these genes, most notably *glnII *and *amtB*, showed a marked loss of transcriptional activity. This unexpected phenomenon was observed consistently for nearly all target genes identified, and in duplicate biological experiments. Therefore a simple model, in which nitrogen starvation leads to an increased affinity of GlnR for its target promoters, which is reversed by sufficient levels of nitrogen, does not adequately explain the observed binding activity. To investigate further, an extra ChIP experiment was carried out 30 min after addition of ammonium chloride (T_60_). However, even at this time point, GlnR was still associated with all target sites identified at T_30_. It would therefore appear that GlnR assumes different forms during the time course. At T_0_, GlnR is present (see Figure 2A) but does not associate with its target promoters, with the exception of *glnA*. At T_30_, GlnR assumes a form with increased affinity for target sequences and association occurs, along with activation of gene expression. At T_45_, DNA binding is unaffected, but transcriptional activity from target genes is abolished, indicating that a transcriptionally inactive form of GlnR is associated with target promoters for at least 30 min after addition of ammonium chloride (Figure 4). As GlnR is a member of the OmpR winged helix-turn-helix family of transcriptional regulators, whose activities are classically modified via phosphorylation [23], it may be that phosphorylation states of GlnR account for the differently active forms. However, many atypical members of the OmpR family that do not undergo phosphorylation have also been characterised [24]. An alternative explanation is that transcriptional activity may still persist at the T45 and T60 time points but stability of target gene mRNA may be severely reduced, leading to a drop in the measured transcript level.

### Promoters enriched in GlnR immunoprecipitates

All of the reproducible GlnR-binding sites (observed in at least two experiments and absent within the control) were located within intergenic regions. A total of thirty six such peaks were identified and Table 3 lists the genes located immediately downstream of the binding sites. Five of these genes; *glnA*, *glnII*, *amtB*, *ureA *and Sven_1860 (SCO2195), are known to be regulated by GlnR in *S. coelicolor *[11,12]. However, none of the promoter regions of the eight other *S. venezuelae *genes that are homologous to GlnR-regulated genes in *S. coelicolor *was enriched in this study.

**Table 3:** Genes directly adjacent to peaks identified in ChIP-chip analysis

Gene ID	*S. coelicolor *homologue	Annotation	Fold change in Transcript level at T30 in *glnR *strain	MEME-Identifed GlnR concensus sites
				
Sven_1863	SCO2198	GSI	-1.2	T**T**A**AC**TTCGAC**G**AA**AC** **GT**C**A**TGCTTGA**G**AA**AT**

Sven_1874	SCO2210	GSII	**-291.6**	**GT**A**AC**ACGGGGT**T**C**AC** **G**CA**AC**CGACGG**G**AA**A**T

Sven_7046	n/a	NRPS cluster	1.1	**G**AA**AC**ACGGGC**G**AA**AC**

Sven_5517	SCO5842	Putative acetyltransferase	1.4	n/a

Sven_6731	SCO5931	Xylanase	-1.5	**G**AA**AC**ATCTTC**G**AA**AC**

Sven_5279	SCO5583	AmtB	**-624.7**	**GT**C**AC**GGCTCC**G**AA**AC**T**T**C**AC**GGTCGC**GT**A**AC**

Sven_4770	n/a	Unknown	-1.1	T**T**A**AC**GCGCAG**G**CA**AC**

Sven_3917/3918	SCO4159/4160	GlnR/putative hydrolase	(n/a)/**30.7**	T**T**C**A**TCCATCC**GT**A**AC**

Sven_6152/6153	n/a	ATP-cassette/cytochrome P450	1.0/-1.2	**GT**T**AC**CCCCAC**GT**A**AC** **GT**T**AC**CGTCGG**GT**C**AC** **GT**A**AC**CGGTCG**GT**A**A**G **GT**G**AC**CCGACG**GT**A**AC**

Sven_6199/6200	n/a	NRPS cluster	1.7/**3.2**	T**T**C**AC**TCCGGC**G**AA**AC** **GT**G**AC**CGCTGA**GT**AG**C** T**T**G**A**TCTCCTG**GT**A**A**A

Sven_2720	SCO2958	Fused RR/uroporphrinogen III synthase	**-13.7**	n/a

Sven_2830	n/a	β-glucan synthesis	-1.4	n/a

Sven_0629/0630	SCO6598/0545	LacI family repressor/secreted protein	**2.5**/1.5	**G**AT**AC**AGGGGG**G**AA**AC**

Sven_0867	SCO1293	N-acetyl glutamate synthase	**-9.1**	T**T**A**AC**CCGTCA**GT**C**AC**

Sven_1634	SCO2008	Branched chain amino acid binding protein	-1.3	A**T**A**AC**AAGACAGTC**AC**

Sven_1677	SCO2026	Glutamate synthase	-1.1	**GT**A**AC**CTGCAC**G**AA**A**T

Sven_5967	SCO3051	FadE acetyl CoA dehydrogenase	-1.3	**G**AC**AC**CCCGAGT**T**A**AC**

Sven_6632	SCO5685	Putative sugar hydrolase	1.4	**GT**T**A**AGTGAAC**GT**C**AC**

Sven_0035		Secreted protein	-1.2	**GT**G**AC**GCCGAG**GT**T**AC**

Sven_2895	SCO3092	Fragment of NADH dehydrogenase	1	T**T**C**AC**AAGGGGTGA**AC**

Sven_2285	SCO4892	Transcriptional regulator	1.1	**G**AA**AC**ACCCTG**GT**A**AC**

Sven_6163	n/a	Unknown	-1.6	n/a

Sven_2694	SCO2937	Transcriptional regulator	-1.4	n/a

Sven_0835	SCO1236	UreA	**-2.5**	T**T**A**AC**GAGCCG**G**AA**A**A

Sven_1338/1339	SCO1721/n/a	Probable serine/threonine protein kinase	1.6/-1.4	**G**AT**AC**ACGGGT**GT**C**AC**

Sven_1860	SCO2195	Unknown	-**4.5**	**GT**C**AC**GCCCTG**GT**A**AC**

Sven_6307	SCO6473	Crotonyl-CoA reductase	-1.7	n/a

Sven_5972/5973	n/a	JadR2/JadR1 (and small orf inbetween)	1.1/-1.7	T**T**A**A**TGGCGGC**GT**CAC T**T**G**AC**CACTTCT**T**G**AC**T**T**G**AC**ACGGAGT**T**G**AC GT**C**A**ACTCCGT**GT**C**AA**

Sven_0812	SCO2423	Secreted protein	1.3	**GT**TT**C**CCGCAA**GT**A**AC**

Sven_1894/1895	SCO2231/2232	Maltose binding protein/transcriptional repressor	1.1/1.2	**G**AC**AC**GCGGAT**GT**A**AC**

Sven_3785	SCO4034	RNA polymerase sigma factor N	-1	n/a

Sven_4759	n/a	Putative peptide transport system secreted peptide-binding protein	1.6	n/a

Sven_1354	SCO1735	Probable secreted lipase	1.3	T**T**T**AC**CGACGC**GT**A**AC GT**C**AC**GCCTTCA**T**GAC

Sven_6299/6300	n/a/SCO6803	Epimerase/acetyl transferase	1.9/**-6.1**	T**T**C**AC**GTGCCC**G**AA**AC**

Sven_7354	SCO7012	Putative binding protein dependent transport protein permease	**-3.7**	n/a

Sven_7089	n/a	Assimilatory nitrate reductase	-1.8	**GT**G**AC**ACAGGT**GT**A**AC**

Peaks were called as present when two or more consecutive probe signals were greater than 2.5 standard deviations above the mean. Duplicate biological experiments were performed for each time point, with independent hybridisations. Peaks listed were present in at least two independent samples. Sections of sequences in bold and underlined are those that match the proposed GlnR consensus sequence.

### The conserved GlnR binding sequence

**Figure 5:**
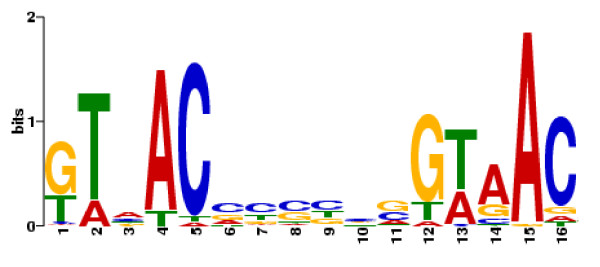
**MEME-derived consensus sequence from GlnR binding regions identified by ChIP-chip analysis**. Consensus GlnR-binding motif generated from 250 bp regions surrounding the peaks identified in ChIP-chip experiments. The genes used to generate the consensus sequence are listed in Table 3.

The identification of a consensus GlnR binding site has proven to be of great value in identifying GlnR-regulated genes [11]. To identify conserved sequences within the promoter regions found to be GlnR-associated *in vivo *in *S. venezuelae*, 250 bp sequences of each promoter region were aligned and a consensus sequence (Figure 5, GTnAC-n_6_-GTnAC) was derived using MEME [20]. Matches present in each promoter sequence are indicated in Table 3. Of the 36 peaks identified, 27 have strong candidate sites from which the MEME motif was derived; however, nine promoter regions lacked a consensus sequence. One such promoter region is that of Sven_2720 encoding a putative transcriptional regulator. Despite lacking the conserved motif, the gene is highly regulated in response to nitrogen availability (induced 21-fold in the wild-type strain by N starvation) in a manner that is exquisitely GlnR-dependent (at T_30 _the level of transcript present in the wild-type strain is 14-fold greater than that in the *glnR *mutant) and GlnR is associated with the promoter region *in vivo*. EMSA experiments with purified GlnR (data not shown) revealed retardation of DNA sequences upstream of *glnA*, *glnII*, *amtB *and Sven_7046, all of which contain the conserved GlnR binding motif. Mobility of the promoter region of Sven_2720 was unaffected in the presence of GlnR.

## Discussion

In this study we combined transcriptomic and ChIP-chip analyses to investigate the genetic control of nitrogen regulation in *S. venezuelae*. We identified a large number of genes within the GlnR regulon but, like others [9], we could not identify a role for the GlnR homologue GlnRII in nitrogen regulation.

The genes identified here can be divided into four categories: i) those that responded to nitrogen availability in a GlnR-dependent manner and where GlnR was associated with the promoter region; ii) those that responded in a GlnR-dependent manner, but showed no GlnR interaction at the promoter and are potentially modulated through another regulatory protein iii) those that responded to nitrogen status independently of GlnR, and iv) those GlnR targets identified by ChIP-chip that showed no response to nitrogen status under the conditions studied. For categories i) and iv), we identified 36 *in vivo *binding sites on the *S. venezuelae *chromosome for the nitrogen responsive transcriptional regulator GlnR, and these cover three major aspects of nitrogen metabolism.

### Primary nitrogen metabolism

As expected, genes that encode proteins known or likely to be directly involved in nitrogen metabolism are highly represented among the GlnR targets. Such functions include the assimilation of nitrogen, either from ammonium (AmtB, GlnK, GlnD, GSI, GSII, glutamate synthase) or from alternative N sources such as urea or nitrate (urease, nitrate reductase). GlnR also regulates production of N-acetyl-glutamate synthase, which, as part of the arginine biosynthesis pathway, synthesises N-acetyl-glutamate from glutamate and acetyl-CoA and whose activity is dependent on cellular nitrogen levels [25]. All of these genes are regulated directly, and possibly solely, by GlnR under the conditions used in these experiments. Two other genes that fall in category i) but have no known function are Sven_1860, encoding a small (71 amino acid) protein, and Sven_2720, encoding a protein with homology to a winged helix-turn-helix motif at the C-terminus and to uroporphyrinogen III synthase at the N-terminus. Based on the regulatory profiles of these genes, they too may be directly involved in primary nitrogen metabolism, and provide interesting targets for future study.

### Nitrogen scavenging

Several genes downstream of GlnR-binding sites (Sven_1634, Sven_4759 and Sven_7354) are predicted to encode periplasmic binding protein (PBP) components of ABC transport systems with substrates such as amino acids and small peptides, suggesting roles in scavenging of alternative nitrogen sources during starvation. Other GlnR-targets encode predicted secreted proteins involved in the degradation of various macromolecules, such as the predicted glycosyl hydrolases, Sven_6731, encoding a xylanase, and Sven_6632, encoding a β-glucosidase, involved in the degradation of plant cell wall polysaccharides [26,27], a predicted peptidase (Sven_6152), and a predicted lipase (Sven_1354). Production of an array of degradative enzymes upon nitrogen starvation may be an attempt to release nitrogen and/or other nutrients, either from plant material or other organisms co-habiting the soil environment in which *Streptomyces *species have evolved.

Many of the genes encoding secreted proteins and PBPs fall into category iv), i.e. GlnR targets in ChIP-chip experiments that were not identified as nitrogen responsive in the transcriptional studies. However, the absence of a nitrogen-dependent transcriptional response may reflect the starvation conditions used in this study. The proteins encoded by these genes are not involved directly in nitrogen metabolism, but may, in some circumstances, perform useful roles under nitrogen-limited conditions. Therefore, for optimal induction, these systems may require additional input signals in addition to nitrogen limitation, such as substrate-dependent induction, that were absent in our experimental conditions.

### Secondary Metabolism

GlnR binds to the intergenic region between the divergently transcribed *jadR1 *and *jadR2 *genes, which encode transcriptional regulators that activate and repress, respectively, expression of the jadomycin biosynthetic genes [28]. Microarray data suggest that transcription of *jadR1 *is activated by GlnR. Expression of *jadR1 *is induced 3.3-fold in response to nitrogen starvation in the wild-type strain and this level is repressed 1.2 fold upon ammonium addition. Consistent with this, levels of *jadR1 *transcription in the *glnR *mutant during nitrogen starvation are 1.7-fold lower than in the wild-type strain (Table 3). Given the antimicrobial activity of jadomycin B [29], GlnR regulation may facilitate induction of expression as a response to nitrogen limitation caused by the presence of competing microorganisms. None of the other genes comprising the jadomycin B gene cluster show any significant changes in expression in response to nitrogen limitation, neither in the wild-type nor the *glnR *mutant, indicating that increased expression of JadR1 alone is not sufficient to activate expression of the cluster. Lack of induction may be due to the action of the repressor JadR2, which is only deactivated upon stress treatments such as heat or ethanol shock [30].

GlnR binding sites were also identified upstream of Sven_7046 and within the intergenic region of Sven_6199 and Sven_6200. These uncharacterised genes are located within regions that have been annotated as potentially encoding non-ribosomal peptide synthetase (NRPS)-like gene clusters. All three were down-regulated in response to nitrogen limitation, but their expression, and that of adjacent genes, was not significantly altered in the *glnR *deletion strain.

### Comparisons with the *S. coelicolor *GlnR regulon

Many of the genes responsive to changes in nitrogen availability in *S. venezuelae *are also regulated by GlnR in *S. coelicolor*. Of the 15 genes identified as GlnR targets in *S. coelicolor *[11], 13 have strong homologues in *S. venezuelae *(genes are considered to be homologous if they are reciprocal top BLAST hits). Four of these 13 genes, namely the *amtB-glnK-glnD *operon and *glnII*, were among the twenty genes showing the largest induction upon N-starvation (Table 1). A further two targets, *ureA *encoding the γ-subunit of urease and Sven_1860 (SCO2195) encoding a small protein of unknown function, also responded significantly (Additional file 2). The remaining seven *S. venezuelae *genes whose homologues are GlnR-regulated in *S. coelicolor *did not pass the filtering criteria, although one, *glnA*, only narrowly failed, being induced 1.9-fold upon N-starvation and repressed 1.7-fold by exogenous ammonium. As observed in *S. coelicolor *[11], induction of *glnA *was notably lower than that of *glnII *(6.5-fold).

The lack of a marked response to N-starvation by some candidate genes could reflect species differences in the GlnR regulon, or differences in experimental conditions. In this study with *S. venezuelae*, N-starvation was achieved by complete removal of any N-source, whereas in *S. coelicolor *N-starvation was achieved by a switch from growth on ammonium to growth on nitrate. *S. venezuelae nirB*, encoding a nitrite reductase subunit, did not respond to N-starvation but the gene was induced in a GlnR-dependent manner in *S. coelicolor*. Hence the presence of nitrate/nitrite may be required for *nirB *induction. Similarly, whereas the complete absence of nitrogen induced *ureA *in *S. venezuelae, S. coelicolor ureA *was repressed upon N-starvation, perhaps indicating a preference for nitrate over urea as a nitrogen source in the latter species. We have not investigated the *in vivo *promoter binding activity of *S. coelicolor *GlnR and there is no published data on this. So it is currently unknown whether *S. coelicolor *GlnR also binds to target promoters *in vivo *under conditions in which transcriptional activation does not occur.

### The GlnR binding site consensus sequence

The MEME algorithm identified a common motif present in twenty-seven of the thirty-six GlnR target regions identified by ChIP-chip, in some cases in multiple copies (Table 3). The motif identified was similar to that proposed for *S. coelicolor *but with some significant differences. The binding site (GlnR box) proposed by Tiffert *et al. *contained an "a-site" (gTnAc) located 6 bps upstream of a "b-site" (GaAAc), giving a consensus sequence of gTnAc-n_6_-GaAAc [11]. Interestingly, recent work by Wang and Zhao [12] identified a novel binding site configuration in the promoter region of *S. coelicolor nasA *(encoding nitrate reductase). GlnR recognition of the *nasA *promoter is mediated by two GTAAC "a-sites" separated by 18 bps, leading the authors to suggest that a "b-site"might not be obligatory. The motif we identified in *S. venezuelae*, GTnAC-n_6_-GTnAC (Figure 5), is essentially two copies of the "a-site" separated by 6 bps. Hence the distinction between the "a" and "b" sites is less well defined in *S. venezuelae *where, although there are several examples of GAnAC occupying the "b-site", GTnAC is much more common (Table 3).

Tiffert *et al. *[11] suggested that GlnR binding may follow the OmpR model, such that the more highly conserved "b" site has a higher affinity for GlnR, whilst the less well conserved "a-site" has a lower affinity. However the sequence derived in their study was based initially on alignments of the strongly GlnR-regulated promoters of *amtB *and *glnA *(as well as SCO1863, which has no homologue in *S. venezuelae*), and may be biased towards a higher affinity "b-site". The promoters of *amtB *and *glnA *in *S. venezuelae *both contain a "b-site" with the sequence GAAAC, and both have two tandem copies of the minimal binding sequence (i.e., GTnAC-n_6_-GTnAC-n_6_-GTnAC-n_6_-GTnAC), an arrangement suggested to be the predominant GlnR-binding site in *S. coelicolor *[11]. This tandem arrangement with a preponderance of GAAAC in the "b" position may be indicative of strong GlnR regulation. However, it is not representative of the majority of GlnR binding sites observed *in vivo *in *S. venezuelae*, where a single occurrence of GTnAC-n_6_-GTnAC is the most common motif.

### Promoters lacking an identifiable GlnR box

Nine of the thirty-six regions identified in ChIP-chip experiments do not contain a GlnR binding motif. Transcription factors binding to non-consensus sequences are a common observation in ChIP-chip studies (reviewed in detail by Wade *et al. *[31]). Examples include well-studied transcription factors such as Fnr of *Escherichia coli *[32] and CtrA of *Caulobacter crescentus *[33]. Likewise, studies of *Bacillus subtilis *SpoA [34] revealed many *in vivo *binding sites that were not bound in an *in vitro *assay. Local changes in DNA topology, or the co-operative interactions of multiple transcriptional factors *in vivo*, may reduce the requirement for the consensus sequence [31] and facilitate binding to non-consensus sites.

As discussed for *nasA *of *S. coelicolor*, GlnR is capable of binding to two distantly separated copies of the GTnAC motif. Inspection of promoter regions that do not contain the full consensus sequence reveals several that possess multiple copies of the GTnAC motif. For example, the Sven_2720 promoter region contains three separate copies of GTnAC separated by regions of thirty nine and thirty three base pairs. Further work is required to establish their possible role in facilitating GlnR binding.

## Conclusions

GlnR is the global nitrogen regulator in actinomycetes and plays a key role in regulating the assimilation and utilisation of nitrogen. This study has extended our knowledge of the GlnR regulon in streptomycetes. It has also indicated a possible link between GlnR and transcription of JadR1, the pathway specific regulator of the jadomycin B cluster, as well as secreted degradative enzymes and several proteins with functions relating to transport. Application of ChIP-chip has provided fresh insight into the DNA sequences to which GlnR binds *in vivo *and has shown that GlnR is able to associate with target promoters in both transcriptionally active and inactive forms.

## Methods

### Strains, primers and plasmids

*S. venezuelae *ATCC 10712 was the wild-type strain used throughout this study. All mutants were generated using PCR targeting( Gust *et al. *[19]). The entire coding region of each gene, including start and stop codons, was replaced with an apramycin resistance cassette amplified from pIJ773 [19]. For generation of the *glnR::apr*^*R *^mutant, the forward primer was 5'-CACCTTGGCCACGCGCGGCAGTCTACGCGGGGTGACCTAATTCCG

GGGATCCGTCGACC-3' and the reverse primer was 5-CGACCGACCGACGGCGGGTCCGGCAGGTGGTGCGCGATGTGTAGGCTGGAGCTGCTTC-3.

For the *glnRII::apr*^*R *^mutant, the forward primer was 5'-TCCGTTCGTTTCTTCGCGCGAAAGAGCTGAGACCTCATGATTCCGGGGATCCGTCGACC-3', and the reverse was 5'-TGGTGTCCAGGACGAGGGCGAAGGCGAACTGACGGATCATGTAGGCTGGAGCTGCTTC-3'.

Primers used for qRT-PCR to measure *amtB *levels were 5-TCCGCCGCCAACACCGGGTTCA-3 and 5-GGCGAGTGCCGGGGTCATCAGC-3, and for *hrdB *levels 5-CATGGCGGACCAGGCCCGAACC-3 and 5-CCTGGAGCATCTGGCGCTGCAC-3.

FLAG-tagging of GlnR was achieved by amplifying a region from genomic DNA that contained the entire GlnR coding sequence excluding the stop codon, along with 264 bp of upstream sequence containing the *glnR *promoter, using the primer pair 5-TATTATAAGCTTGTGGGCTATTCTCCT-3 and 5-CCTACCGGCAGGTCGCACTGTGGC-3. The amplicon was blunt-ended using *Pfu *polymerase (Invitrogen) and cloned into the *StuI *site of pIJ10500 (courtesy of C den Hengst, John Innes Centre, Norwich), a modified version of the integrative pMS82 vector [21] containing a *Streptomyces *codon usage-optimised triple FLAG epitope cassette, thereby creating a C-terminally tagged GlnR protein expressed from its native promoter in construct pIJ12248.

### Growth conditions

For all liquid culture experiments *S. venezuelae *was grown in 30 ml batches of Evans defined minimal media [18]. The nitrogen source used was 30 mM NH_4_Cl, unless stated otherwise. To transfer *S. venezuelae *between media types, the culture was transferred to a falcon tube and very briefly centrifuged to form a pellet. Media was decanted and the pellet resuspended in the alternative media. For growth on solid media, Evans was supplemented with agarose at a final concentration of 2% w/v.

### RNA extraction

At each experimental time point 10 ml of culture were centrifuged briefly to pellet the mycelium which was rapidly frozen under liquid nitrogen. Frozen pellets were ground using a pestle and mortar containing liquid nitrogen. The broken cells were mixed with 1.5 ml of TRI^® ^Reagent (Sigma) and left at room temperature for 5 min before addition of 300 μl chloroform. The mixture was vortexed briefly then incubated at room temperature for 2 min. Samples were centrifuged for 10 min at 13000 rpm in a bench top microfuge. The aqueous phase, containing RNA, was harvested and RNA was purified using an RNeasy^® ^Mini Kit (Qiagen) as per manufacturer's instructions, including the recommended on-column DNase digestion. The final elution step was carried out with 50 μl of nuclease-free water (Qiagen).

### qRT-PCR analysis

Specific primers for *amtB *and *hrdB *were designed using the Primer3 web-based tool [35]. RNA (5 μg) was treated with amplification grade RNase-free DNaseI (Invitrogen) according to the manufacturer's instructions. The resulting RNA was used as template for cDNA synthesis in a 20 μl reaction using Superscript III First Strand Synthesis Supermix (Invitrogen) according to manufacturer's instructions. PCR was performed at 25°C for 10 min, 42°C for 120 min, 50°C for 30 min, 55°C for 30 min and 85°C for 5 min. Samples were diluted x100 in Tris-EDTA (10 mM, pH 8.0), and 2.5 μl were used for quantitative SYBR Greener qPCR supermix (Invitrogen) reactions according to manufacturer's instructions. 200 nM of forward and reverse primers were used in each 25 μl reaction. PCR was performed in a BioRad Chromo4 machine at 50°C for 2 min, 95°C for 10 min, followed by 40 cycles of 95°C for 15 s and 58°C for 60 s. Identical reactions were performed using sequential dilutions of genomic DNA to generate a standard curve for each primer pair. Biological experiments were performed in triplicate, the results were analysed using Opticon 2 Monitor software (MJ Research) and values were normalized to levels of *hrdB *expression.

### Affymetrix GeneChip hybridization and data collection for expression studies

Purified total RNA (10 μg) was used as the template for production of cDNA that was subsequently labelled and fragmented for hybridisation to Affymetrix *Streptomyces *diS_div712a GeneChip arrays as described previously by Hesketh *et al *[36]. Hybridizations were performed according to protocols provided by the manufacturer in a Hybridization Oven model 640 (Affymetrix.). The GeneChips were washed and stained with streptavidin-phycoerythrin using GeneChip fluidics workstation model 450, and then scanned with a GeneArray Scanner Model 3000 7G.

### Data analysis

Expression data were imported into GeneSpring 9.0 (Agilent Technologies), normalised using the Robust Multichip Average algorithm (RMA), converted to log_2 _values and normalised per gene to the median.

Microarray data have been deposited in the ArrayExpress datatbase, under the accession number E-MEXP-2684.

Two-way ANOVA was performed in GeneSpring using the parametric test option with a false discovery rate of *P *< 0.01 or *P *< 0.05, and assuming variances to be equal. *P *values were corrected using the Benjamini and Hochberg false discovery rate multiple testing correction procedure.

### Western blotting

*S. venezuelae *strains were grown to an OD_600 _of ~ 0.6; 10 ml of culture was briefly centrifuged to form a pellet, which was resuspended in 1.5 ml of SP buffer and sonicated on ice. Multiple 15 s bursts of sonication at 10 kHz were performed with 60 s intervals between bursts, until the suspension became clear. Cell debris was removed by centrifugation. Protein concentrations of cell fractions were determined using the Bio-Rad protein assay system using bovine serum albumin as a standard. In all cases, 5 μg of total protein was separated by SDS-PAGE (15% polyacrylamide). After transfer to a nitrocellulose membrane (Hybond ECL nitrocellulose membrane; Amersham), the proteins were reacted with monoclonal ANTI-FLAG^® ^M2 antibody (Sigma).

### Chromatin-immunoprecipitation

#### Cell preparation and cross-linking

Fifty millilitre cultures of *S. venezuelae *were grown to an OD_600 _of ~ 0.6 and formaldehyde (Sigma) was added to a final concentration of 1%. Cross linking was allowed to proceed for 30 min of continued incubation at 30°C. The addition of glycine, at a final concentration of 125 mM, halted the cross-linking. Cells were briefly centrifuged to form a pellet and washed twice with ice-cold PBS. The pellet was resuspended in 750 μl of lysis buffer (10 mM Tris-HCl pH 8.0, 50 mM NaCl, 10 mg/ml lysozyme, supplemented with 1 pellet of Roche complete mini EDTA-free protease inhibitor per 10 ml of buffer) and incubated for 25 min at 25°C. Samples were placed on ice for 2 min after addition of 750 μl of IP buffer (100 mM Tris-HCl pH 8.0, 250 mM NaCl, 0.5% Triton X-100, 0.1% SDS, also supplemented with protease inhibitor). Samples at this point resembled a slurry that was sonicated repeatedly at 10 kHz in 15 s bursts; sub-fractions were taken at frequent intervals, extracted twice with phenol/chloroform and run on agarose gels to check fragment size. Sonication was complete, typically after 7-8 cycles, when fragments were between 300 and 1000 bp in length, centred on 500 bp. Debris was removed from the sonicated extracts by centrifugation at maximum speed in a bench top microfuge for 10 min at 4°C and supernatant fluid was retained. At this point 25 μl of the extract was stored to be used as the control "total DNA" whilst the remainder, ~725 μl, was used in immunoprecipitation.

### Immunoprecipitation, reversal of cross-linking, and elution of DNA

Initial pre-clearance of the extracts was performed by incubation for 1 h at 4°C on a rotating wheel after addition of 1/10 volume of a 50% Protein A-sepharose slurry (Sigma) equilibrated in IP buffer. The mixture was centrifuged to pellet the beads, the cleared extract harvested and mixed with 5 μl monoclonal ANTI-FLAG^® ^M2 antibody (Sigma). Incubation was then continued overnight. A 1/10 volume of a fresh 50% Protein A-sepharose slurry was added and samples incubated for a further 4 h at 4°C before centrifugation for 5 min at 3500 rpm in a microfuge to harvest the bead-antibody-chromatin complex. This was washed once in 0.5x IP buffer and twice in IP buffer each for 15 min with gentle agitation. Elution of DNA was performed by addition of 150 μl of IP elution buffer (50 mM Tris-HCl pH 7.6, 10 mM EDTA, 1% SDS) to the beads, as well as to 10 μl of the "total DNA" control, and incubation at 65°C overnight. Beads were pelleted by centrifugation and the supernatant harvested, treated with 2 μl of 10 mg/ml Proteinase K (Roche) and twice extracted with phenol chloroform. Finally, DNA was purified using the Qiagen QiaQuick according to manufacturer's instructions.

### Microarray design, labelling, and hybridization

Custom made microarray slides, consisting of 44,000 60-mer oligonucleotide probes covering the entire *S. venezuelae *genome, were designed and produced by Oxford Gene Technologies (Oxford, UK). Labelling of control total DNA and immunoprecipitated DNA, with Cy5 and Cy3 respectively, as well as hybridisation, washing and scanning were performed by Oxford Gene Technologies according to their standard protocols (http://www.ogt.co.uk ) previously described in detail for *E. coli *[37].

### Data analysis

Cy3/Cy5 ratios were calculated for each probe. Ratios were then plotted against genome position. A peak in the plot representing a protein binding site was scored as present when firstly, two consecutive probes gave a ratio that was 2.5 standard deviations above the mean calculated across all probes in at least two of the six experiments, and secondly when this peak was absent in the control sample, derived by immuno-precipitation of a culture of *S. venezuelae *carrying an empty vector instead of the FLAG-tagged GlnR construct. The ChIP-chip data have been deposited in the ArrayExpress database, under the accession number E-MEXP-2933.

## List of abbreviations

ChIP: chromatin immuno-precipitation; qRT-PCR: quantitative reverse-transcriptase polymerase chain reaction; IP: immunoprecipitation; PBP: periplasmic binding protein; NRPS: non-ribosomal peptide synthetase.

## Authors' contributions

STP carried out all experimental work, participated in design of the study, analysed data and drafted the manuscript. GC programmed analytical tools for interpretation of ChIP-chip and aided the analysis of sequence data. MB participated in design and coordination of the study and helped to draft the manuscript. MM conceived of the study, and participated in its design and coordination and helped to draft the manuscript. All authors read and approved the final manuscript.

## Supplementary Material

Additional file 1 **Microarray expression profiles of *amtB-glnK-glnD*, *glnA *and *glnII *over the wild-type, *glnR *and *glnRII *mutant time courses**. Expression is the average of three biological replicates and normalised intensity is plotted on a log_2 _scale. Diagram adapted from GeneSpring 9.0 (Agilent). Click here for file

Additional file 2 **Full list of genes induced >2 upon nitrogen starvation, that are also repressed by ammonium**. Click here for file

Additional file 3 **Genes induced >2 fold by nitrogen starvation and repressed by ammonium in the wild-type strain, but non-responsive in the *glnR *mutant strain**. Click here for file
