# Supplementary material for: Genome-wide analysis of the role of GlnR in Streptomyces venezuelae provides new insights into global nitrogen regulation in actinomycetes
Source: BMC Genomics. 2011 Apr 4;12:175. doi: 10.1186/1471-2164-12-175 (PMC3087709; doi:10.1186/1471-2164-12-175)

Pullan *et al*. Additional File 1.

Microarray expression profiles of *amtB-glnK-glnD*, *glnA* and *glnII* over the wild-type, *glnR* and *glnRII* mutant time courses.
Expression is the average of three biological replicates and normalised intensity is plotted on a log_2 scale. Diagram adapted from GeneSpring 9.0 (Agilent).

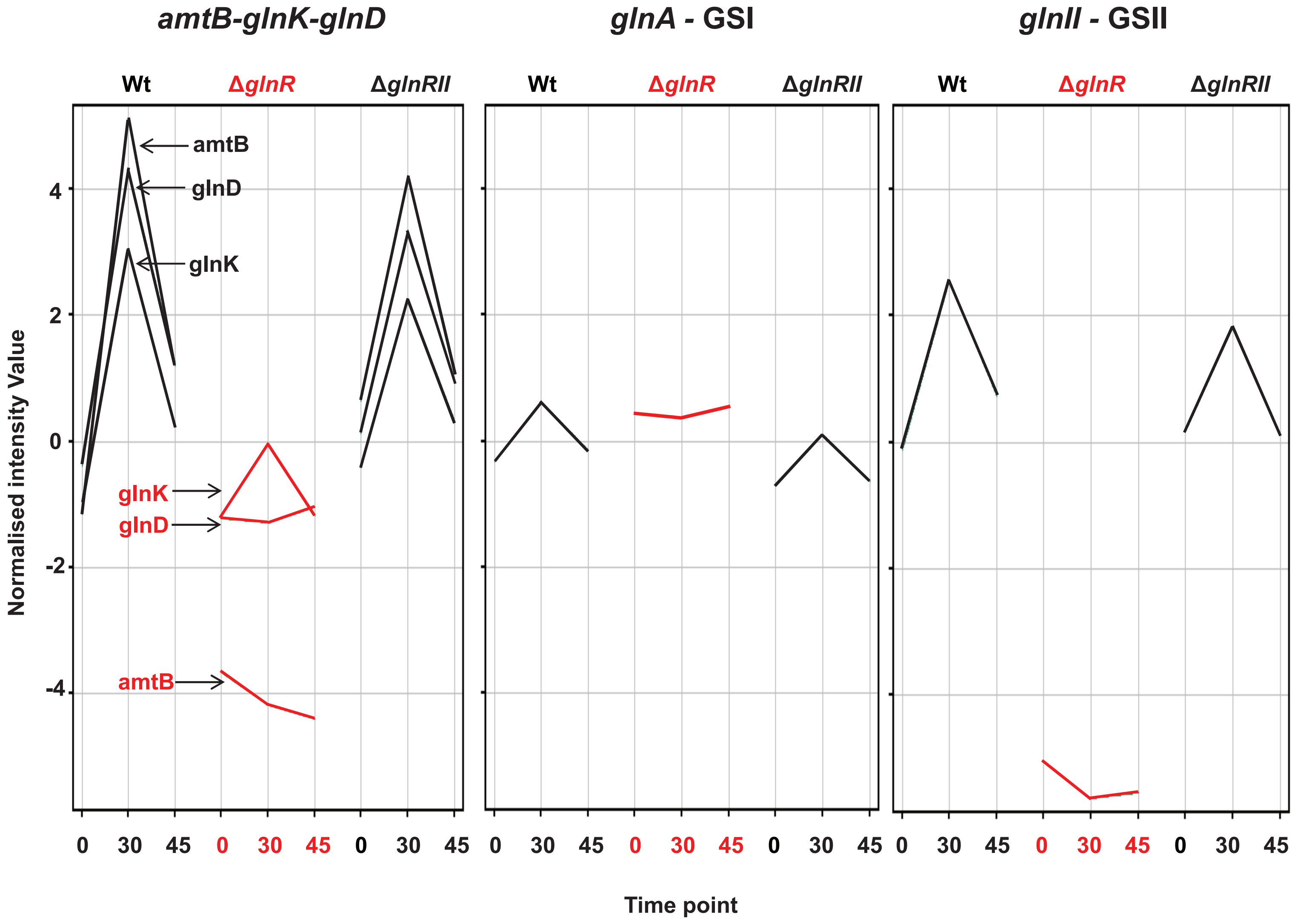

Supplement: Additional file 1 — Microarray expression profiles of amtB-glnK-glnD, glnA and glnII over the wild-type, glnR and glnRII mutant time courses. Expression is the average of three biological replicates and normalised intensity is plotted on a log2 scale. Diagram adapted from GeneSpring 9.0 (Agilent). [file 1471-2164-12-175-S1.PDF]
