# Supplementary material for: Genome-wide analysis of the role of GlnR in Streptomyces venezuelae provides new insights into global nitrogen regulation in actinomycetes
Source: BMC Genomics. 2011 Apr 4;12:175. doi: 10.1186/1471-2164-12-175 (PMC3087709; doi:10.1186/1471-2164-12-175)
Supplement: Additional file 2 — Full list of genes induced >2 upon nitrogen starvation, that are also repressed by ammonium. [file 1471-2164-12-175-S2.PDF]

Pullan *et al*. Additional file 2.

Full list of genes induced >2 upon nitrogen starvation, that are also repressed by ammonium.

Gene ID	*S. coelicolor* homologue	Annotation	Fold Induction in WT at	Fold Repression in Wt at T45	Corrected p-value Time
Sven_5279	SCO5583	ammonium transporter *amtB*	76.4	15.0	3.91E-05
Sven_5281	SCO5585	PII uridylyltransferase *glnD*	26.0	8.3	1.34E-05
Sven_2720	SCO2958	putative transcriptional regulator	21.6	6.0	8.24E-03
Sven_5280	SCO5584	nitrogen regulatory protein PII *glnK*	16.0	6.8	4.90E-04
Sven_2606	SCO2816	conserved hypothetical protein	15.4	3.9	8.92E-06
Sven_0745	SCO1118	putative integral membrane protein	13.6	9.9	1.72E-04
Sven_4809	SCO5163	unknown	12.5	3.7	3.58E-07
Sven_0933	-	unknown	11.7	7.2	4.03E-02
Sven_6300	SCO6803	putative acetyltransferase	9.8	2.9	3.20E-02
Sven_4564	SCO4896	putative transport integral membrane protein	9.2	7.3	4.32E-06
Sven_1595	SCO1963	putative integral membrane export protein	8.7	2.2	1.38E-05
Sven_3456	SCO6809	putative integral membrane transport protein	7.9	6.2	9.06E-04
Sven_1523	-	unknown	7.7	4.5	5.71E-04
Sven_2474	SCO5348	putative excisionase	7.4	2.7	2.70E-03
Sven_3334	SCO3564	putative Na+/H+ antiporter	7.3	4.6	1.42E-06
Sven_0739	SCO1109	putative oxidoreductase	7.2	4.4	2.51E-08
Sven_3030	SCO3185	putative Na+/H+ antiporter	7.1	4.1	1.27E-07
Sven_0867	SCO1293	unknown	6.7	5.2	1.08E-04
Sven_1874	SCO2210	glutamine synthetase II *glnII*	6.5	3.6	2.52E-02
Sven_3001	SCO3167	putative tetR-family transcriptional regulator	6.4	7.5	1.71E-06
Sven_5427	SCO5772	unknown	6.4	2.2	4.25E-02
Sven_1172	SCO1578	acetylglutamate kinase	6.4	10.1	4.55E-05
Sven_6667	SCO4935	putative integral membrane protein	6.0	3.1	3.20E-04
Sven_3458	SCO3696	putative transcriptional regulator	5.9	2.7	1.69E-02
Sven_3383	-	unknown	5.9	9.9	3.39E-04
Sven_0779	-	unknown	5.8	3.5	2.82E-02
Sven_4820	-	unknown	5.7	2.7	5.17E-04
Sven_6135	-	unknown	5.7	4.2	1.94E-03

Sven_2419	SCO2636	unknown	5.7	2.0	9.93E-03
Sven_3474	-	unknown	5.6	4.5	1.82E-07
Sven_1169	SCO1572	putative secreted protein	5.5	8.7	1.18E-03
Sven_3292	SCO4995	putative membrane protein	5.4	7.0	3.45E-03
Sven_4152	SCO4337	putative integral membrane efflux protein	5.4	2.8	3.58E-07
Sven_6071	-	unknown	5.4	11.5	2.21E-02
Sven_1173	SCO1579	putative glutamate N-acetyltransferase	5.1	6.5	4.41E-05
Sven_2176	SCO2362	unknown	5.0	3.9	4.29E-02
Sven_2575	SCO2788	unknown	5.0	7.1	1.70E-08
Sven_4575	SCO4908	putative RNA polymerase sigma factor	4.9	3.2	8.91E-06
Sven_2018	-	unknown	4.8	3.3	3.86E-03
Sven_4151	SCO4336	putative marR-family protein	4.6	3.1	7.99E-08
Sven_1168	SCO1570	argininosuccinate lyase	4.6	6.0	2.27E-04
Sven_3057	SCO3202	RNA polymerase principal sigma factor	4.6	2.3	2.86E-08
Sven_0157	SCO4297	putative oxidoreduxtase	4.6	2.4	8.40E-06
Sven_7188	SCO0393	putative transferase	4.5	13.6	1.50E-02
Sven_2128	-	unknown	4.5	8.2	3.65E-02
Sven_2932	-	unknown	4.4	5.0	1.27E-02
Sven_5471	SCO5796	conserved hypothetical protein	4.4	2.8	5.97E-09
Sven_5899	-	unknown	4.4	2.2	1.45E-07
Sven_2177	SCO2363	putative ATP/GTP-binding protein	4.3	3.4	3.56E-04
Sven_1171	SCO1577	acetonitrile aminotransferase	4.2	7.4	3.84E-05
Sven_3410	SCO4407	unknown	4.2	3.5	2.17E-06
Sven_5938	SCO6152	small hydrophobic protein	4.1	6.6	3.07E-02
Sven_3957	SCO4208	putative integral membrane transport protein	4.1	2.3	1.27E-02
Sven_0712	-	unknown	4.1	2.1	3.29E-02
Sven_2692	-	unknown	4.1	4.2	1.27E-07
Sven_6456	SCO6771	putative small hydrophobic secreted protein	4.0	2.5	2.44E-04
Sven_2576	SCO2789	glucosamine-fructose-6-phosphate aminotransferase	4.0	8.6	1.27E-07
Sven_1176	-	unknown	4.0	8.2	1.12E-04
Sven_1860	SCO2195	unknown	3.9	3.2	3.38E-02
Sven_2693	-	unknown	3.9	5.1	1.09E-04
Sven_7105	SCO7255	conserved hypothetical protein	3.7	3.0	1.38E-04
Sven_0332	-	unknown	3.7	4.7	6.84E-04
Sven_0738	SCO1108	putative regulatory protein	3.7	3.2	6.03E-03
Sven_0837	SCO0856	putative integral membrane protein	3.6	2.1	1.89E-02

Sven_3000	SCO3166	putative membrane transport protein	3.6	3.3	1.84E-06
Sven_1174	SCO1580	N-acetyl-gamma-glutamyl-phosphate reductase	3.5	6.8	5.05E-04
Sven_3472	-	unknown	3.5	2.1	2.10E-04
Sven_0977	-	unknown	3.5	2.1	2.33E-05
Sven_4582	-	unknown	3.4	2.5	2.51E-08
Sven_5010	SCO5358	putative membrane protein	3.4	3.4	4.68E-09
Sven_3772	SCO4020	putative two component system response regulator	3.3	4.8	1.56E-03
Sven_1170	SCO1576	arginine repressor	3.3	4.1	9.87E-06
Sven_6863	SCO3050	unknown	3.3	2.2	2.23E-07
Sven_1522	SCO1875	putative secreted penicillin binding protein	3.3	2.4	1.57E-03
Sven_3462	SCO6836	putative ArsR-family transcriptional regulator	3.3	2.5	2.45E-06
Sven_3421	-	unknown	3.2	2.4	7.09E-05
Sven_1213	SCO1617	putative integral membrane protein	3.1	2.7	2.55E-03
Sven_1166	SCO1568	putative tetR-family transcriptional regulator	3.1	2.9	1.24E-02
Sven_3915	SCO4157	putative protease	3.0	2.6	1.33E-04
Sven_3940	-	unknown	2.9	2.5	6.49E-06
Sven_3248	SCO3396	putative membrane protein	2.8	2.6	3.36E-06
Sven_3463	SCO6835	putative arsenate reductase	2.8	3.1	8.52E-03
Sven_0868	SCO1294	putative cystathionine gamma-synthase	2.8	2.7	1.80E-03
Sven_3432	SCO3670	heat shock protein	2.7	3.3	1.35E-04
Sven_1778	SCO2117	putative anthranilate synthase	2.7	2.3	3.72E-03
Sven_6718	-	unknown	2.7	5.0	3.93E-02
Sven_0252	-	unknown	2.6	5.1	2.83E-02
Sven_1038	SCO1441	3,4-dihydroxy-2-butanone 4-phosphate synthase	2.6	3.1	3.74E-04
Sven_4583	SCO3737	putative lipoprotein	2.6	2.7	3.80E-02
Sven_4823	SCO6111	putative peptide ABC transporter ATP-binding protein	2.6	3.8	4.05E-02
Sven_1928	SCO2241	probable glutamine synthetase	2.6	2.3	3.21E-03
Sven_2382	SCO2601	putative integral membrane protein	2.6	2.0	1.69E-02
Sven_3061	SCO3206	putative transmembrane efflux protein	2.5	2.2	4.52E-05
Sven_1175	SCO7036	argininosuccinate synthase	2.5	5.5	1.58E-03
Sven_0999	SCO0443	conserved hypothetical protein	2.5	3.0	3.77E-02
Sven_1165	SCO1567	putative transmembrane-transport protein	2.5	5.0	2.38E-02
Sven_3384	-	unknown	2.5	3.1	1.79E-05
Sven_3062	SCO3207	putative tetR-family transcriptional regulator	2.5	2.6	1.27E-07
Sven_3595	-	unknown	2.4	5.3	1.55E-02
Sven_4346	SCO4655	DNA-directed RNA polymerase beta' chain	2.3	2.8	3.01E-06

Sven_1039	SCO1442	putative integral membrane protein	2.3	2.1	2.23E-04
Sven_2086	-	unknown	2.3	3.8	3.31E-02
Sven_5663	-	unknown	2.3	3.0	8.13E-03
Sven_0177	SCO3915	putative transmembrane efflux protein	2.3	2.6	1.37E-03
Sven_3473	SCO4450	putative tetR-family transcriptional regulator	2.2	2.4	1.46E-02
Sven_3433	SCO3671	heat shock protein 70	2.2	2.4	2.78E-07
Sven_3459	SCP1.270	unknown	2.2	4.2	5.79E-03
Sven_6938	-	putative tetR family transcriptional regulatory protein	2.2	2.4	1.52E-02
Sven_2559	-	conserved hypothetical protein	2.2	4.0	1.81E-02
Sven_5904	-	unknown	2.2	3.8	8.61E-04
Sven_5592	SCO6842	unknown	2.2	2.1	2.99E-02
Sven_2479	-		2.2	2.5	4.50E-02
Sven_0835	SCO1236	urease gamma subunit *ureA*	2.2	2.2	1.57E-04
Sven_5343	-	unknown	2.1	3.9	9.70E-03
Sven_7333	-	unknown	2.1	2.3	3.58E-03
Sven_4858	SCO5205	unknown	2.0	2.4	8.87E-04
