# Supplementary material for: Genome-wide analysis of the role of GlnR in Streptomyces venezuelae provides new insights into global nitrogen regulation in actinomycetes
Source: BMC Genomics. 2011 Apr 4;12:175. doi: 10.1186/1471-2164-12-175 (PMC3087709; doi:10.1186/1471-2164-12-175)
Supplement: Additional file 3 — Genes induced >2 fold by nitrogen starvation and repressed by ammonium in the wild-type strain, but non-responsive in the glnR mutant strain. [file 1471-2164-12-175-S3.PDF]

Pullan *et al.* Additional File 3.

Genes induced >2 fold by nitrogen starvation and repressed by ammonium in the wild-type strain, but non-responsive in the *glnR* mutant strain.

Gene ID	*S. coelicolor* homologue	Annotation
Sven_5279	SCO5583	ammonium transporter, *amtB*
Sven_5281	SCO5585	PII uridylyltransferase, *glnD*
Sven_2720	SCO2958	putative transcriptional regulator
Sven_2606	SCO2816	conserved hypothetical protein
Sven_6300	SCO6803	putative acetyltransferase
Sven_4564	SCO4896	putative integral membrane protein
Sven_1595	SCO1963	putative integral membrane export protein
Sven_2474	SCO5348	putative excisionase
Sven_3334	SCO3564	putative Na+/H+ antiporter
Sven_0867	SCO1293	unknown
Sven_1874	SCO2210	glutamine synthetase II
Sven_5427	SCO5772	unknown
Sven_1172	SCO1578	acetylglutamate kinase
Sven_3383	-	unknown
Sven_0779	-	unknown
Sven_6135	-	unknown
Sven_2419	SCO2636	unknown
Sven_1169	SCO1572	putative secreted protein
Sven_4152	SCO4337	putative integral membrane efflux protein
Sven_1173	SCO1579	putative glutamate N-acetyltransferase
Sven_2176	SCO2362	unknown
Sven_4151	SCO4336	putative marR-family protein
Sven_1168	SCO1570	argininosuccinate lyase
Sven_3057	SCO3202	RNA polymerase principal sigma factor
Sven_2932	-	unknown
Sven_5899	-	unknown
Sven_1171	SCO1577	acetonitrile aminotransferase
Sven_3410	SCO4407	unknown
Sven_3957	SCO4208	putative integral membrane transport protein
Sven_1176	-	unknown
Sven_1860	SCO2195	unknown
Sven_7105	SCO7255	conserved hypothetical protein
Sven_0738	SCO1108	putative regulatory protein
Sven_0837	SCO0856	putative integral membrane protein
Sven_3000	SCO3166	putative membrane transport protein
Sven_1174	SCO1580	N-acetyl-gamma-glutamyl-phosphate reductase
Sven_0977	-	unknown
Sven_4582	-	unknown
Sven_1170	SCO1576	arginine repressor
Sven_6863	SCO3050	unknown
Sven_3462	SCO6836	putative ArsR-family transcriptional regulator
Sven_3421	-	unknown
Sven_1213	SCO1617	putative integral membrane protein
Sven_1166	SCO1568	putative tetR-family transcriptional regulator
Sven_3940	-	unknown
Sven_3463	SCO6835	putative arsenate reductase
Sven_0868	SCO1294	putative cystathionine gamma-synthase

Sven_1778	SCO2117	putative anthranilate synthase
Sven_6718	-	unknown
Sven_0252	-	unknown
Sven_4583	SCO3737	putative lipoprotein
Sven_4823	SCO6111	putative peptide ABC transporter ATP-binding protein
Sven_1928	SCO2241	probable glutamine synthetase
Sven_2382	SCO2601	putative integral membrane protein
Sven_3061	SCO3206	putative transmembrane efflux protein
Sven_1175	SCO7036	argininosuccinate synthase
Sven_3062	SCO3207	putative tetR-family transcriptional regulator (fragment)
Sven_3595	-	unknown
Sven_4346	SCO4655	DNA-directed RNA polymerase beta' chain (fragment)
Sven_1039	SCO1442	putative integral membrane protein
Sven_2086	-	unknown
Sven_5663	-	unknown
Sven_3473	SCO4450	putative tetR-family transcriptional regulator
Sven_6938	-	putative tetR family transcriptional regulatory protein
Sven_2559	-	conserved hypothetical protein
Sven_5904	-	unknown
Sven_5592	SCO6842	unknown
Sven_0835	SCO1236	urease gamma subunit *ureA*
Sven_5343	-	unknown
Sven_7333	-	unknown
